# Comprehensive Transcriptomic and Physiological Insights into the Response of Root Growth Dynamics During the Germination of Diverse Sesame Varieties to Heat Stress

**DOI:** 10.3390/cimb46120794

**Published:** 2024-11-22

**Authors:** Xiaoyu Su, Chunming Li, Yongliang Yu, Lei Li, Lina Wang, Dandan Lu, Yulong Zhao, Yao Sun, Zhengwei Tan, Huizhen Liang

**Affiliations:** 1 Institute of Chinese Herbal Medicines, Henan Academy of Agricultural Sciences, Zhengzhou 450002, China; suxiaoyu_2014@163.com (X.S.); lchm1212@163.com (C.L.); yyl790721@126.com (Y.Y.); 15136189572@163.com (L.L.); hnndlina@163.com (L.W.); ludandan0710@163.com (D.L.); zhaoyulong2009@163.com (Y.Z.); ruth_3912834@163.com (Y.S.); 2 Provincial Key Laboratory of Conservation and Utilization of Traditional Chinese Medicine Resources, Zhengzhou 450002, China

**Keywords:** root growth, RNA sequencing, regulatory mechanism, heat stress, sesame

## Abstract

Heat stress constitutes a serious threat to sesame (*Sesamum indicum* L.). Root development during seed germination plays an essential role in plant growth and development. Nevertheless, the regulatory mechanisms underlying heat stress remain poorly understood. In this study, two sesame varieties differing in leaf heat tolerance (Zheng Taizhi 3 (heat-tolerant) and SP19 (heat-sensitive)) have been employed to investigate the impact of heat stress on root growth during germination. The results showed that heat stress significantly reduced the radicle length by 35.71% and 67.02% in Zheng Taizhi 3 and SP19, respectively, while germination rates remained unchanged. In addition, heat stress induced oxidative stress, as evidenced by increased reactive oxygen species (ROS) production, malondialdehyde (MDA) content, and reduced indole-3-acetic acid (IAA) content, accompanied by enhanced antioxidant enzyme activities, including those of superoxide dismutase (SOD), peroxidase (POD), and catalase (CAT), and the abscisic acid (ABA) content significantly increased in both varieties. However, the oxidation resistance in the roots of Zheng Taizhi 3 was enhanced compared to that of SP19 under heat stress, while IAA production was maintained and ABA content was reduced. A comparative transcriptome analysis identified 6164 and 6933 differentially expressed genes (DEGs) in Zheng Taizhi 3 and SP19, respectively, with 4346 overlapping DEGs. These DEGs included those related to stress tolerance, such as heat-shock proteins (HSPs), the antioxidant defense system, hormone signal transduction, and the biosynthetic pathway of phenylpropanoid. These findings provide insights into the physiological and molecular mechanisms underlying the adaptation of sesame to heat stress, which could inform breeding strategies for developing heat-tolerant sesame varieties.

## 1. Introduction

Global warming has resulted in more and more frequent extreme high-temperature weather, which has seriously affected the production of crops [1]. Heat stress can cause metabolic disorders in plant cells, affecting plant photosynthetic efficiency, enzyme activity, protein synthesis, and causing oxidative stress, which can lead to plant death in severe cases [2]. Sesame is an important economic crop in China, which has the growth habit of being thermophilic but not heat-resistant. The optimum temperature for the growth of sesame is 24–30 °C. When the ambient temperature is higher than 40 °C, the germination is affected, and the leaves and stems of sesame will wilt in a short amount of time, or even experience tissue death [3].

In the long-term process of natural selection and artificial domestication, plants have gradually evolved various complex and efficient regulatory mechanisms to resist and adapt to high-temperature stress. Sesame is sensitive to high temperatures, and its molecular mechanism of heat resistance is rarely studied. For instance, Su et al. analyzed two sesame varieties after high-temperature treatment by transcriptome, and found that heat-resistant sesame varieties mainly improved the heat resistance of sesame by affecting the expression of genes related to plant hormone signal transduction and heat-shock protein (HSP) gene expression [4]. HSPs are the main type of functional protein induced by heat stress, which act as a molecular chaperone to prevent protein misfolding and aggregation at high temperatures [5]. At present, HSP genes that can improve plant heat tolerance have been identified in many crops such as tomato (*Solanum lycopersicum* L.), maize (*Zea mays* L.), and rice (*Oryza sativa* L.) [6]. Although some research has been conducted on the response of sesame to elevated temperatures, the majority of studies have focused on sesame leaves [3,4,7]. Conversely, there is a scarcity of research investigating the response of sesame roots to heat stress.

Both roots and leaves are sensitive to heat stress, but roots are usually no less or even more sensitive to heat stress than leaves [8,9,10]. The relationship between the growth status of root systems in plants exposed to high temperatures and their heat tolerance has been investigated in a number of studies. For example, in creeping bent grass, researchers found that the better performance of L-93 (heat-tolerant) under heat stress was due largely to the root growth rather than to the Penncross (heat-sensitive) [11]. Jiang et al. investigated the impact of heat stress on root growth in tall fescue and perennial ryegrass. Their findings indicated that heat-induced damage to root growth represents a significant limitation on the growth of cool-season grasses during the summer [12]. Heat, in addition to causing physiological and morphological changes in plants, can have a significant impact on the internal environment’s homeostasis, resulting in distortions in cellular organization and, ultimately, plant growth. For example, the RNA-seq data for ginseng (*Panax ginseng* C. A. Meyer) indicated that the repression of root growth in response to heat stress is closely associated with stress-related hormones and the signaling pathway involving reactive oxygen species (ROS) [13]. Additionally, elevated temperatures can influence the synthesis of free amino acids in roots, potentially conferring resilience to environmental stress [14]. Overall, these findings highlight the pivotal role of root development in plant adaptation to heat stress. Thus, the study of the molecular mechanism of heat tolerance in sesame roots is critical for the breeding of heat-tolerant sesame varieties and the improvement of sesame yield and quality under heat stress.

In order to further investigate the molecular mechanisms of heat tolerance in root growth during the germination of sesame under heat-stressed environments, this study utilized two different sesame varieties, whose leaves exhibited differential tolerances to elevated temperatures. The impact of heat stress on root growth, antioxidant protective enzyme activities, reactive oxygen species (ROS), and hydrogen peroxide (H_2_O_2_) levels during sesame germination was determined. Subsequently, the heat stress-related genes were identified through the use of RNA-Seq and real-time quantitative PCR (qRT-PCR) techniques. These findings indicate the possibility of further investigation into the role of candidate genes in the response of sesame to heat stress, as well as a new perspective on the molecular regulatory mechanisms of sesame in high temperatures.

## 2. Materials and Methods

### 2.1. Plant Material, Heat Treatment, and Sample Collection

Two sesame varieties (the tolerant Zheng Taizhi 3 and the sensitive SP19) of seeds were soaked for 12 h in a growth chamber, with a temperature of (25 ± 2) °C, a relative humidity of (60 ± 5)%, a photoperiod of 12 h/12 h, and a light intensity of 1000 µmol photons m^–2^ s^–1^. The sesame seeds that were healthy and uniform were chosen and randomly divided into two groups. One group was moved into a growth chamber with a 25 °C (day/night) temperature as the control (CK). The other group was moved into a growth chamber with a 40 °C (day/night) temperature as HT stress. Roots from individual plants under C (normal) and H (heat) conditions were collected 6 d after treatment, frozen immediately in liquid nitrogen, and stored at −80 °C. Roots from at least three individual plants grown under C or H conditions were pooled as one replicate, with at least three replicates for each group.

### 2.2. Phenotypic and Physiological Measurements

For the assessment of heat stress in sesame seedlings, root length was recorded. The electrical conductivity was measured using a DDS-307 conductivity meter (INESA Scientific Instrument Co., Ltd., Shanghai, China). The relative electrical conductivity (REC) was determined as described previously [15]. Briefly, 0.5 g of roots are soaked in 10 mL of deionized water for 3 h, after which the conductivity is measured and recorded as R1. After 30 min, the solution is boiled, and the conductivity is measured and recorded as R2. The REC was calculated by the formula of R1/R2 × 100%. MDA content was estimated following the method described by Kulshreshtha et al. (1983) [16]. O_2_^·−^ production rate was determined using the hydroxylamine oxidation method as described by Elstner et al. (1976) [17]. The H_2_O_2_ content was measured using previously reported methods [18].

For the assay of antioxidant enzyme activities, the total SOD, POD, and CAT activities of sesame roots were determined as described previously [19]. Briefly, 0.2 g of roots were homogenized in a mortar and pestle with 4 mL of ice-cold extraction buffer (100 mM potassium phosphate buffer, pH 7.0, 100 µM EDTA). The homogenate was centrifuged at 16,000× *g* for 20 min. The supernatant fraction was used as the crude extract for enzyme activity. Total SOD activity was determined by measuring its ability to inhibit the photochemical reduction of nitro blue tetrazolium chloride (NBT). Total CAT activity was measured according to the decrease of H_2_O_2_ monitored at 240 nm and quantified by its molar extinction coefficient (36 mM^−1^ cm^−1^). The result was expressed as mmol H_2_O_2_ min^−1^ g^−1^ FW (fresh weight). Total POD activity was determined by following the decrease in ascorbate at 290 nm (extinction coefficient of 2.8 mM^−1^ cm^−1^) for 1 min and the results were expressed in mmol H_2_O_2_ min^−1^ g^−1^ FW, taking into consideration that 2 M of ascorbate are required for the reduction of 1 M of H_2_O_2_.

### 2.3. Quantification of IAA and ABA by Enzyme-Linked Immunosorbent Assay (ELISA) Kit

Phytohormone extraction was conducted in accordance with the methodology described previously [20]. In summary, 0.1 g of fresh roots were ground in liquid nitrogen and extracted using 1 mL of ice-cold 80% methanol (*v*/*v*). After incubating the extracts overnight at 4 °C, they were centrifuged at 12,000× *g* for 15 min. Then, the supernatants were measured as IAA and ABA contents using ELISA kits, following the manufacturer’s instructions (Suzhou Comin Biological Co., Ltd., Suzhou, China).

### 2.4. RNA-Seq and Data Analysis

The samples were frozen in liquid nitrogen and then stored at −80 °C. RNA was extracted from samples with a Trizol reagent(Yeasen Biotechnology (Shanghai) Co., Ltd., Shanghai, China). RNA quantity and integrity were measured with agarose gel electrophoresis and Agilent 2100 (Agilent Technologies, Palo Alto, CA, USA), respectively. The mRNA sequencing libraries were constructed using the HieffNGS^®^ Ultima Dual mode mRNA Library Prep Kit (Yeasen Biotechnology (Shanghai) Co., Ltd., Shanghai, China) and sequenced on Illumina HiSeq^TM^ 4000 (Illumina, San Diego, CA, USA).

Raw data were subjected to fastp for removing low-quality data. Clean data (clean reads) were aligned to the reference genome (assembly_S_indicum_v1.0) using HISAT 2.2.1 software (http://ccb.jhu.edu/software/hisat2/index.shtml, accessed on 22 October 2024). The expression of each gene was normalized to fragments per kilobase of transcript per million mapped reads (FPKM) to compare among different samples. The R package “DESeq2” was used to identify differently expressed genes (DEGs) with relative fold changes (FCs) above 2 and a false discovery rate (FDR) lower than 0.05. Then, we performed the analysis of Gene Ontology (GO) enrichment and the Kyoto Encyclopedia of Genes and Genomes (KEGG).

### 2.5. Validation of RNA-Seq Results by qRT-PCR

In order to verify the reliability of the sequencing data, 12 unigenes involved in key pathways were selected for qRT-PCR verification from common DEGs between two varieties. The primers used for qRT-PCR are listed in Table S5. Gene expression levels were determined by the 2^−∆∆Ct^ approach, with *SiTUB* (ncbi_105175801) used as the endogenous control gene.

### 2.6. Statistical Analysis

All experiments were carried out using three biological replicates. The data were analyzed using a one-way ANOVA, and results with *p* values less than 0.05 were considered significant. Results were represented as means ± standard deviation (SD) of 3 biological replicates.

## 3. Results

### 3.1. The Roots of Zheng Taizhi 3 Are More Thermal Resistant than SP19 During Germination

**Figure 1:**
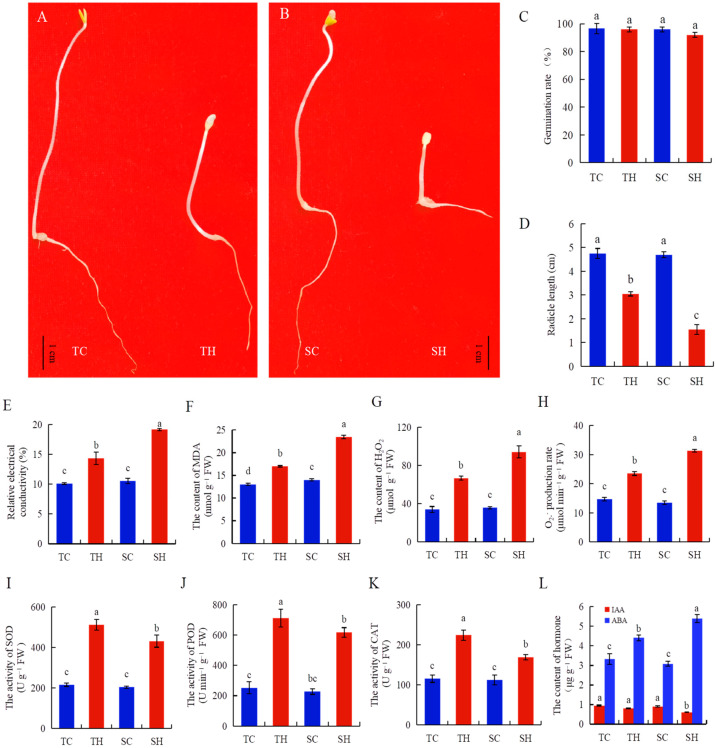
Analysis of the phenotype and physiological effects of heat stress on two contrasting sesame varieties, Zheng Taizhi 3 (heat-tolerant) and SP19 (heat-sensitive). Phenotypes of Zheng Taizhi 3 and SP19 before heat stress treatment (left) and after heat stress treatment (right) (**A**,**B**); germination rate (**C**), radicle length (**D**), relative electronical conductivity (**E**), MDA content (**F**), H_2_O_2_ content (**G**), O_2_^·−^ production rate (**H**), SOD activity (**I**), POD activity (**J**), CAT activity (**K**), IAA and ABA content (**L**) were analyzed in Zheng Taizhi 3 and SP19 under heat stress. TH, heat treatment of the tolerant variety, Zhengtaizhi 3; TC, control of the tolerant variety, Zhengtaizhi 3; SH, heat treatment of the sensitive variety, SP19; SC, control of the sensitive variety, SP19. Different letters indicate significant differences between groups at *p* < 0.05 according to Duncan’s multiple range test.

For assessing the effects of heat stress on root growth during germination, certain phenotypic and physiological characteristics were examined in root samples of normal condition (C) and heat treatment condition (H) sesame plants. The results showed that the root growth of both Zheng Taizhi 3 and SP19 was severely inhibited during the germination under heat stress, with the degree of root inhibition being more pronounced in SP19 than in Zheng Taizhi 3 (Figure 1A,B). However, there was no significant difference in the germination rate of the two sesame varieties under high-temperature stress (Figure 1C). Following a six-day germination period under normal conditions, the average radicle length of Zhengtai 3 was measured at 4.73 cm, while that of SP19 was 4.62 cm. However, following a six-day period of germination under conditions of elevated temperature, it was recorded that the average radicle length of Zheng Tai 3 was 3.05 cm, while that of SP19 was 1.62 cm. Their average radicle lengths were inhibited by 35.71% and 67.02%, respectively (Figure 1D). After heat treatment, REC, O_2_^·−^ production rate, H_2_O_2_, and MDA contents of Zheng Taizhi 3 and SP19 were increased by 41.66% and 81.82%, 60.50% and 132.43%, 96.44% and 163.94%, and 30.31% and 67.30% under heat stress, respectively, compared to the control group (Figure 1E–H). The SOD, POD, and CAT activities of Zheng Taizhi 3 and SP19 were increased by 137.13% and 111.23%, 182.33% and 171.45%, and 93.67% and 49.92% under heat stress, respectively, compared to the control group (Figure 1I–K). The contents of the IAA of Zheng Taizhi 3 and SP19 were decreased by 15.49% and 32.84%, while the ABA contents were increased by 32.57% and 75.03%, under heat stress, respectively, compared to the control group (Figure 1L).

### 3.2. Overview of RNA-Seq

To uncover the molecular mechanism of sesame adaptation to heat stress, RNA-seq was performed on C and H plants. The PCA plot indicated that the replicates of different treatments were clustered into distinct groups, suggesting the good reliability of data (Figure S1A). The correlation heatmap shows that the correlation coefficients within the group are all greater than 0.7 (Figure S1B). To confirm the reliability of RNA-seq, 12 unigenes involved in key pathways were selected from common DEGs between two varieties. Importantly, similar changes in expression were observed for the 12 unigenes, consistent with the results of RNA-seq (Figure S2). These results indicated that the RNA-seq data were of high quality and could be used in later analyses.

**Figure 2:**
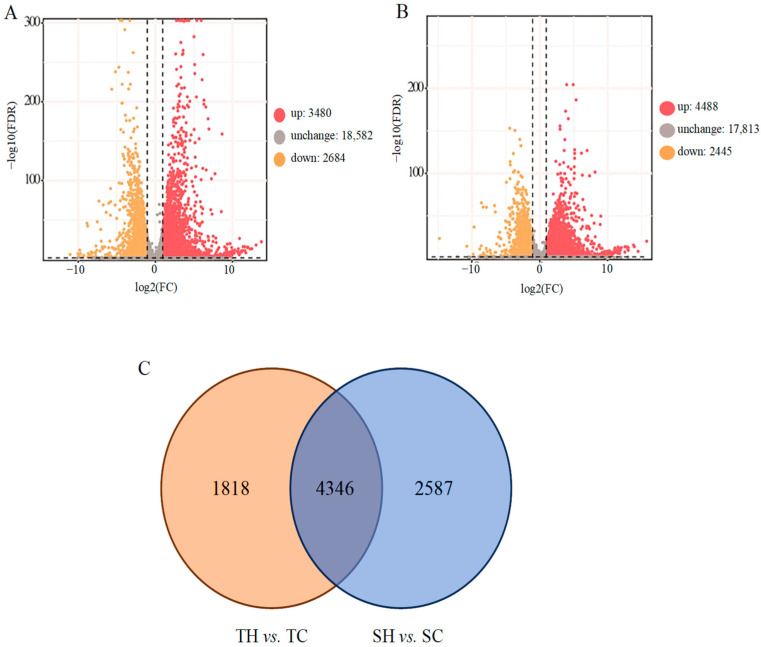
Transcriptomic responses to heat stress in sesame. (**A**) Volcano plot of TH vs. TC; (**B**) volcano plot of SH vs. SC; C, column chart of the DEGs; (**C**) Venn diagrams displaying DEGs in diverse treatments. TH, heat treatment of the tolerant variety, Zhengtaizhi 3; TC, control of the tolerant variety, Zhengtaizhi 3; SH, heat treatment of the sensitive variety, SP19; SC, control of the sensitive variety, SP19.

**Figure 3:**
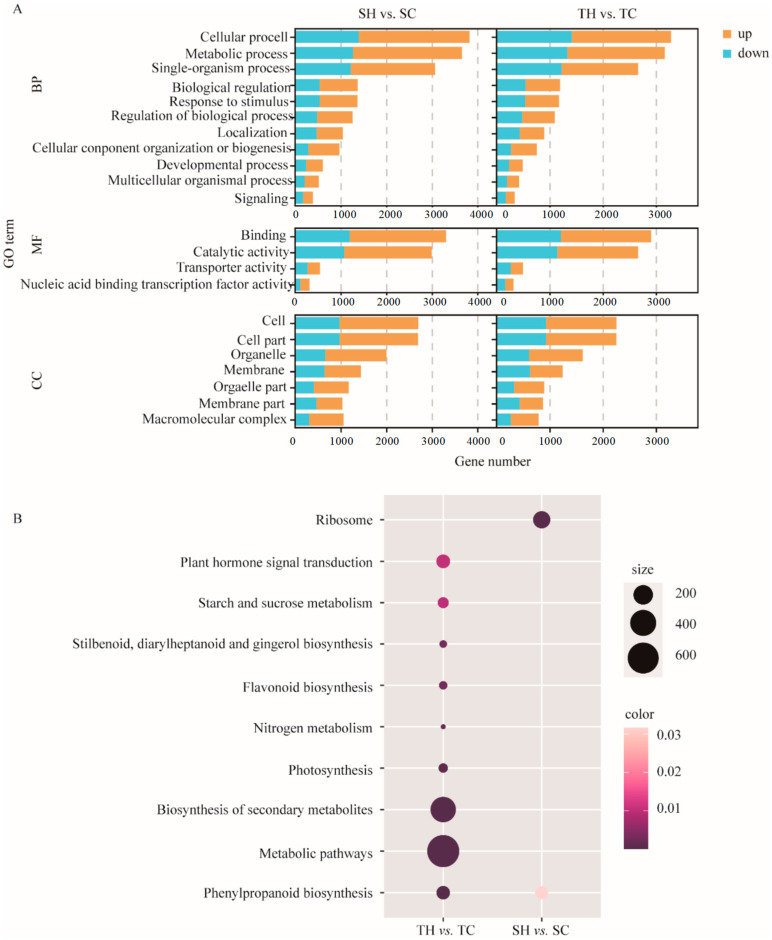
Transcriptome enrichment analysis. (**A**) GO analysis of DEGs in different groups. (**B**) KEGG analysis of DEGs in diverse comparisons. Circle size and color suggest the number of DEGs associated with each KEGG pathway and *p*-values, respectively. TH, heat treatment of the tolerant variety, Zhengtaizhi 3; TC, control of the tolerant variety, Zhengtaizhi 3; SH, heat treatment of the sensitive variety, SP19; SC, control of the sensitive variety, SP19.

A total of 6164 DEGs were obtained from TH vs. TC, including 3480 that were up-regulated and 2684 that were down-regulated. A total of 6933 DEGs, including 4488 that were up-regulated and 2445 that were down-regulated, were found in SH vs. SC (Figure 2A,B). A total of 1818 DEGs existed in TH vs. TC, and 2587 DEGs were detected in SH vs. SC. A total of 4346 DEGs were commonly shared in TH vs. TC and SH vs. SC (Figure 2C). To further the analysis of which DEGs are involved in which biological processes, GO and KEGG enrichment analyses were performed (Figure 3A). After GO enrichment analysis, DEGs were enriched in molecular function, cell component, and biological processes (Figure S3). The top three in the biological process were the “metabolic process”, “cellular process”, and “single-organism process” (Figure S3). The cell components were mainly distributed into “cell”, “cell part”, “organelle”, “membrane”, “organelle part”, “membrane part”, and “macromolecular complex” (Figure S3). The most differential expressed genes in molecular function were “binding”, “catalytic activity”, “transporter activity”, and “nucleic acid binding transcription factor activity” (Figure S3). The KEGG analysis results showed that “phenylpropanoid biosynthesis” was commonly enriched in both groups (Figure 3B). In addition, DEGs were specifically enriched in pathways related to “metabolic pathways”, the “biosynthesis of secondary metabolites”, “photosynthesis”, “nitrogen metabolism”, “flavonoid biosynthesis”, “stilbenoid diarylheptanoid and gingerol biosynthesis”, “starch and sucrose metabolism”, and “plant hormone signal transduction” in TH vs. TC, but “ribosome” was only enriched by SH vs. SC (Figure 3B).

### 3.3. Identification and Analysis of Antioxidant Enzyme Related Genes

**Figure 4:**
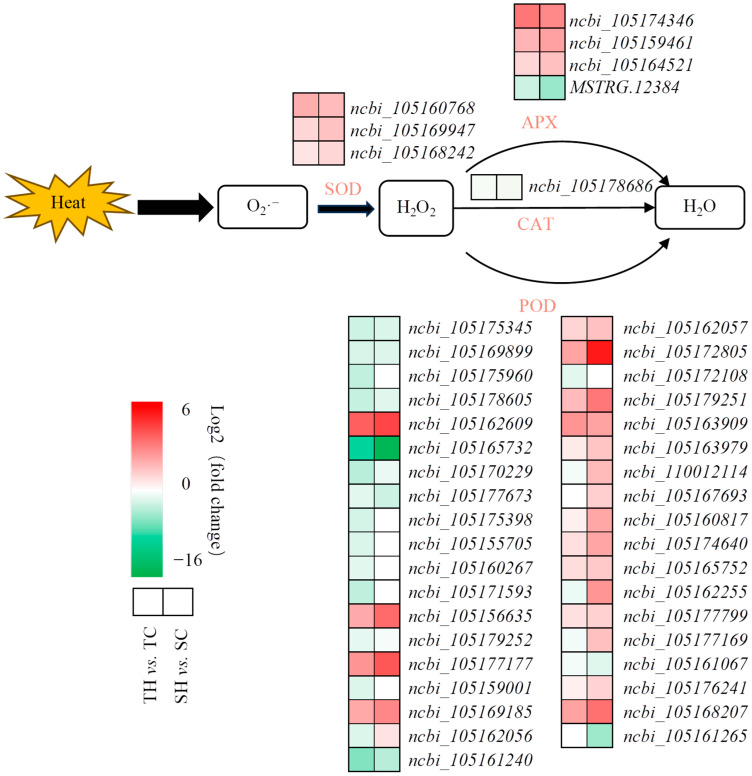
Expression of antioxidant enzyme system related enzyme genes in sesame root under heat stress.

In this study, a total of 45 DEGs related to the plant antioxidant enzyme were identified (Figure 4, Table S1). One DEG in Zheng Taizhi 3 (one up-regulated) and three DEGs in SP19 (three up-regulated) encoding SOD were identified. Four DEGs in Zheng Taizhi 3 (three up-regulated and one down-regulated) and four DEGs in SP19 (three up-regulated and one down-regulated) encoding APX were identified. Twenty-four DEGs in Zheng Taizhi 3 (8 up-regulated and 16 down-regulated) and 25 DEGs (17 up-regulated and 8 down-regulated) encoding POD were identified. In addition, only one DEG encoding CAT was down-regulated in SP19.

### 3.4. Identification and Analysis of Phenylpropanoid Biosynthesis-Related Genes

**Figure 5:**
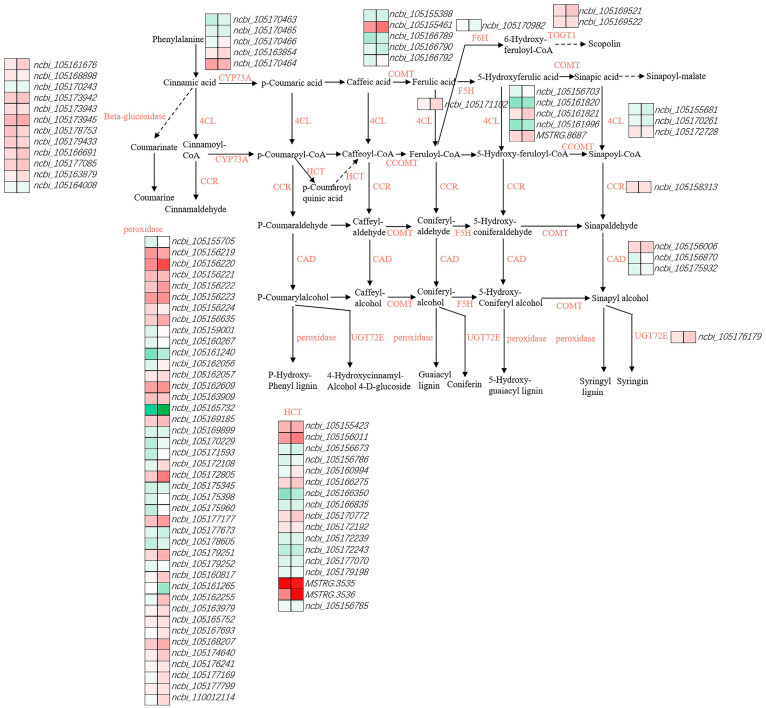
Expression of phenylpropanoid biosynthesis-related enzyme genes in sesame root under high heat stress.

In this study, a total of 102 DEGs related to phenylpropanoid biosynthesis were identified (Figure 5, Table S2). Three DEGs in Zhengtaizhi 3 (one up-regulated and two down-regulated) and one DEG in SP19 (up-regulated) encoding CAD were identified. Thirty DEGs in Zhengtaizhi 3 (14 up-regulated and 16 down-regulated) and 29 (22 up-regulated and 7 down-regulated) encoding peroxidase (POD) were identified. Three DEGs in Zhengtaizhi 3 (three down-regulated) and four DEGs in SP19 (two up-regulated and two down-regulated) encoding trans-cinnamate 4-monooxygenase (CYP73A) were identified. Five DEGs in Zhengtaizhi 3 (two up-regulated and three down-regulated) and four DEGs in SP19 (two up-regulated and two down-regulated) encoding caffeoyl-CoA O-methyltransferase were identified. DEGs encoding beta-glucosidase, cinnamoyl-CoA reductase (CCR), scopoletin glucosyltransferase (TOGT1), coniferyl-alcohol glucosyltransferase (UGT72E) and ferulate-5-hydroxylase (F5H) were mainly up-regulated in the high-temperature group. Sixteen DEGs in Zhengtaizhi 3 (seven up-regulated and nine down-regulated) and thirteen DEGs in SP19 (six up-regulated and seven down-regulated) encoding shikimate O-hydroxycinnamoyltransferase (HCT) were identified. Also, DEGs encoding 4-coumarate-CoA ligase (4CL), caffeic acid 3-O-methyltransferase (COMT) and feruloyl-CoA 6-hydroxylase (F6H) were mainly down-regulated in the high-temperature group.

### 3.5. Identification and Analysis of HSP-Encoding Genes

**Figure 6:**
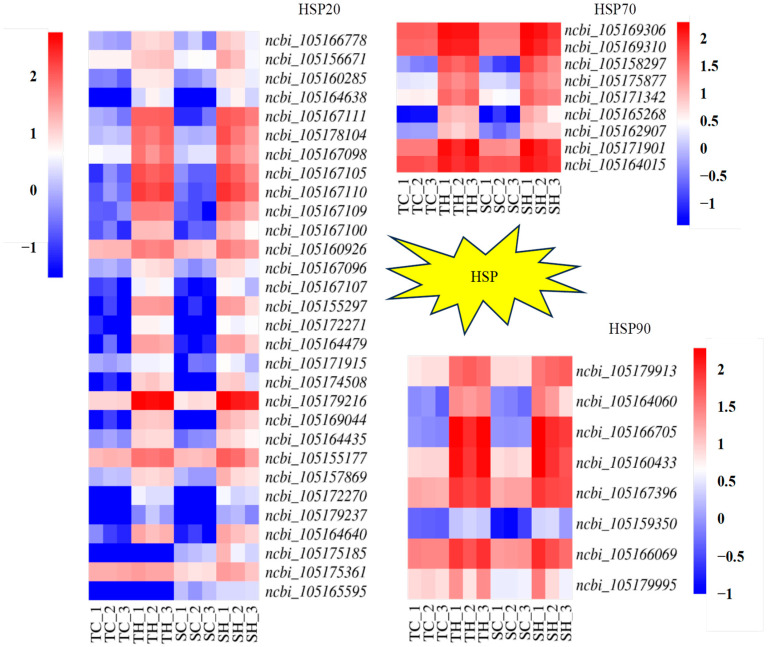
Comparison of heat-responsive HSP family encoded genes’ expression in heat-sensitive (S) and heat-tolerant (T) sesame varieties.

By gene functional annotations, 47 HSP-encoding genes were identified in the two comparison groups, all of which were up-regulated (Figure 6, Table S3). Among these 47 genes, 43 were identified in both varieties; 4 of these genes were specifically up-regulated in the heat-sensitive variety. There were 30 genes belonging to the HSP20 family, including 27 genes in the heat-tolerant variety and 30 up-regulated genes in the heat-sensitive variety, 9 genes belonging to the HSP70 family, including 8 genes in the heat-tolerant variety and 9 up-regulated genes in the heat-sensitive variety, and 8 genes belonging to the HSP90 family in both varieties.

### 3.6. Identification and Analysis of Signal Transduction-Related Genes

**Figure 7:**
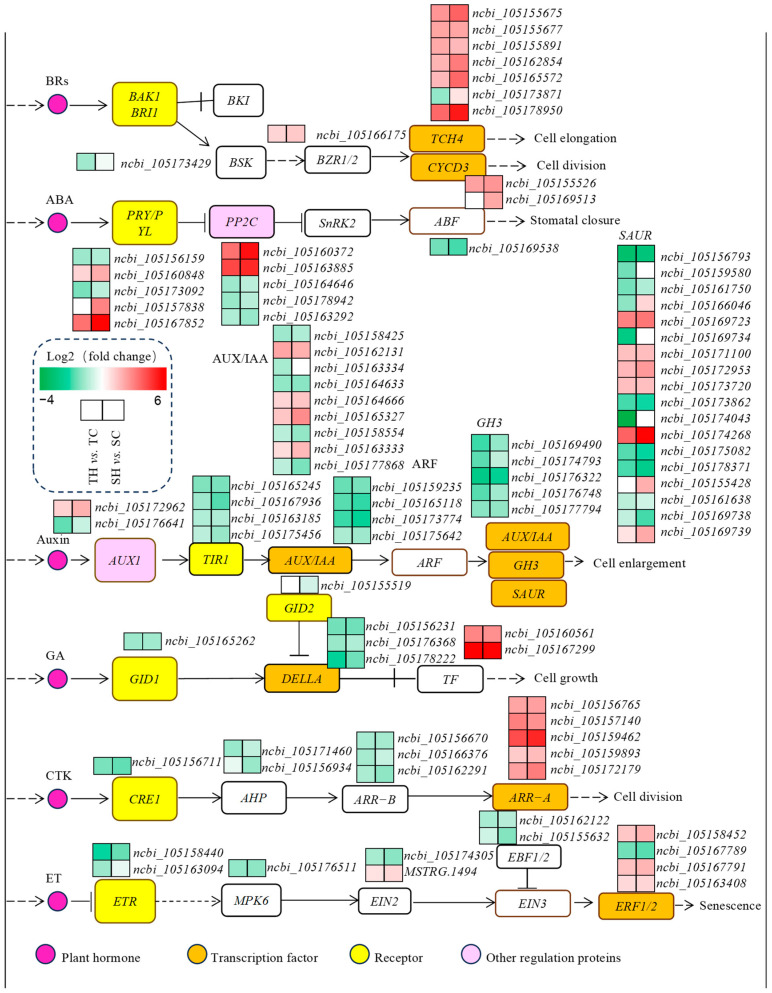
The response in plant hormone signal transduction to high-temperature treatment.

In this study, a total of 109 DEGs related to the plant hormone signal transduction pathway were identified (Figure 7, Table S4). Eight DEGs in Zheng Taizhi 3 (three up-regulated and five down-regulated) and seven DEGs in SP19 (five up-regulated and two down-regulated) were identified to be involved in abscisic acid (ABA) signal transduction pathways. Thirty-two DEGs in Zheng Taizhi 3 (9 up-regulated and 23 down-regulated) and 24 DEGs in SP19 (9 up-regulated and 15 down-regulated) were involved in auxin signal transduction pathways. Nine DEGs in Zheng Taizhi 3 (five up-regulated and four down-regulated) and eight DEGs SP19 in (five up-regulated and three down-regulated) were involved in cytokinin signal transduction pathways. Six DEGs in Zheng Taizhi 3 (two up-regulated and four down-regulated) and four DEGs in SP19 (two up-regulated and two down-regulated) were involved in gibberellin signal transduction pathways. In addition, 11 ethylene (ETH)-related DEGs (2 up-regulated and 6 down-regulated in Zheng Taizhi 3, 2 down-regulated and 2 up-regulated in SP19), 11 brassinolide (BR)-related DEGs (8 up-regulated and 2 down-regulated in Zheng Taizhi 3; 6 up-regulated in SP19), 10 jasmonic acid (JA)-related DEGs (7 down-regulated in Zheng Taizhi 3, and one up-regulated and 3 down-regulated in SP19), and 6 salicylic acid (SA)-related DEGs (all down-regulated in Zheng Taizhi 3 and SP19) were identified, respectively.

## 4. Discussion

High temperatures could significantly inhibit plant growth and agricultural productivity. Sesame is a warm season oil crop and a promising candidate for studying plant response to high-temperature stress (HTS). However, there has been a lack of research investigating the response of the sesame root system to heat stress. The objective of this study was to investigate the response of the root growth dynamics during the germination of diverse sesame varieties to heat stress using transcriptome analysis. The analysis of transcriptional data to elucidate the molecular mechanisms by which heat stress affects the growth of the root system, and to summarize the sesame germination process, is a significant and distinctive aspect of this study.

### 4.1. Physiological Response of Root Growth Dynamics During the Germination of Diverse Sesame Varieties to Heat Stress

In general, heat stress significantly impairs seed germination and metabolism through a variety of mechanisms [21]. Generally speaking, plants grow under normal conditions and can resist excessive ROS accumulation through their own antioxidant defense system to promote normal growth. However, long-term heat stress can inhibit the normal growth of crops, destroy the relatively stable ROS in plants, and lead to the proliferation of H_2_O_2_, and O_2_^·−^. Excessive amounts of ROS cannot be removed in a timely manner, which will accelerate the process of membrane lipid peroxidation, reduce the integrity of the membrane system, damage proteins, and ultimately lead to physiological and biochemical metabolic disorders in plants and inhibit plant growth and development. In order to eliminate excess ROS in the body, plants develop antioxidant systems to alleviate the toxic effects of ROS. SOD can clear superoxide anion free radicals and produce the disproportionate product H_2_O_2_. H_2_O_2_ is catalyzed by POD, CAT, and APX to form water [22].

In this study, we investigated the impact of heat stress on the root systems of two sesame varieties. The heat tolerance of the roots of Zheng Taizhi 3 was found to be significantly higher than that of SP109 (Figure 1A,B), a finding that is also consistent with the heat tolerance of the leaves of both varieties [4]. Moreover, compared to SP19, the roots of Zheng Taizhi 3 exhibited higher activities of SOD, POD, and CAT, and lower REC and contents of MDA, H_2_O_2_, and O_2_^·−^ under heat stress (Figure 1). These results are consistent with previous research results for the leaves of Zheng Taizhi 3 and SP19 [4], indicating that Taizhi 3 inhibits heat-induced membrane damage by increasing antioxidant protective enzymes’ activities to reduce ROS accumulation in heat-stressed sesame roots compared with SP19.

As a key endogenous factor in the plant stress response, plant hormones achieve fine regulation of the stress response through complex networks of different hormone signaling pathways and their cross-signaling [23]. IAA is essential for plant cell polarity development and cell elongation. A large number of studies have confirmed that plants under stress conditions delay growth by reducing growth-promoting hormones, thereby resisting adverse environmental impacts. Our study also found that the content of IAA in sesame roots decreased significantly under heat stress (Figure 1L), which was consistent with the change in IAA content in rice under heat stress [24]. As a stress hormone, ABA plays an important role in the plant response to various stresses. The results of this study showed that the high temperature induced the rapid accumulation of ABA in sesame roots, with a more pronounced accumulation observed in heat-sensitive varieties (Figure 1L). These suggested that the increase in ABA content was closely related to heat tolerance but may also have a detrimental effect on root growth and development, which was consistent with previous studies [24,25].

### 4.2. Phenylpropanoid Biosynthesis of the Root Growth Dynamics During the Germination of Diverse Sesame Varieties in Response to Heat Stress

Phenylpropanoids contribute significantly to plants’ response towards biotic and abiotic stresses [26]. Previous studies have demonstrated that phenylpropanoid biosynthesis is enhanced when plants are subjected to biotic or abiotic stresses, and many of these compounds have been shown to contribute to the plant resistance to pathogens and stress [27]. Phenylpropanoids are also involved in the production of lignin, a complex organic polymer that strengthens plant cell walls and is indigestible by most organisms, thereby providing an additional level of defense [28]. The high level of phenylpropanoids provides resistance to biotic and abiotic stress [29]. The biosynthetic pathway of phenylpropanoids is induced in plants under environmental stress to cope with these harmful conditions. In this study, the phenylpropanoid biosynthesis pathway was enriched in both TH vs. TC and SH vs. SC. Here, we found 102 DEGs that belong to the phenylpropanoid biosynthesis pathway (Figure 5, Table S2). Interestingly, phenylpropanoid biosynthesis was the only enriched common pathway in DEGs between the two varieties. These results suggest that phenylpropanoid biosynthesis may be involved in the high temperature response of sesame. We found that most genes belonging to beta-glucosidase, *CCR*, *TOGT1*, *UGT72E*, and *F5H* were up-regulated in the high temperature compared with the CK (Figure 5, Table S2). The up-regulation of the expression of these key genes involved in the phenylpropane biosynthesis pathway may lead to the promotion of lignin synthesis. These results may be related to the fact that the high temperature group was more active than the CK and constantly produce new cell walls.

### 4.3. HSP of the Root Growth Dynamics During the Germination of Diverse Sesame Varieties in Response to Heat Stress

Many studies have shown that HSP produced under high-temperature heat shock can improve plant heat resistance. When plants are subjected to environmental stress, HSPs act as molecular chaperones to promote the folding, processing, and transport of newly synthesized proteins and the renaturation of intracellular denatured protein [30]. These mechanisms promote the repair of the damaged proteins and the maintenance of cell survival under abiotic stress. Reports have shown that the over-expression of HSP genes in plants could increase tolerance to HTS by protecting chlorophyll synthesis [31]. Many studies have confirmed the expression of *HSPs* induced by high temperatures in a variety of plant species. For example, Sedaghatmehr et al. found that an sHSP encoded by the *AtHSP21* gene in *Arabidopsis thaliana* accumulated rapidly after HTS and retained the function of “heat memory”, enabling rapid protective functions when similar stress is encountered in the future [32]. HSPs are grouped into different classes based on their molecular weights in kilodaltons (kDa), such as *HSP100*, *HSP90*, *HSP70*, *HSP60*, *HSP40*, and *HSP20* (small HSP, sHSP), respectively [33,34]. In this study, 47 HSP genes were identified in response to HTS among the two sesame varieties; all of these genes were up-regulated (Figure 6, Table S3). Moreover, the *HSPs* of SP19 increased more than those of Zheng Taizhi 3 (Figure 6, Table S3), which suggested that SP19 mobilized heat-shock proteins to a greater extent to survive under high-temperature stress.

### 4.4. Plant Hormone Signal Transduction of the Root Growth Dynamics During the Germination of Diverse Sesame Varieties in Reponse to Heat Stress

Plant hormones are key regulatory factors in response to stress, playing an important role in the defense mechanisms of plants against environmental stimuli. Under heat stress, the plant hormone signal transduction pathway is enriched with 93 DEGs. Several DEGs are also involved in signal transduction pathways for other hormones, including ABA, cytokinin, gibberellin, and ethylene.

Plant auxin plays an important role in regulating plant growth and development [35], and has been reported to play a positive role in the reaction to high temperatures [36]. Under high-temperature stress, exogenous auxin can alleviate the damage of high temperatures to rice and to facilitate the maintenance of good yields [37]. Auxin responds to HTS by regulating the expression levels of downstream genes induced by the auxin signaling pathway. Several components of the auxin signal transduction pathway, including AUX1, transport response inhibitor 1, Aux/IAA, and auxin response factors (ARFs), have been identified [38]. In this study, five *GH3*, four *TIR*, and four *ARF homologs* were down-regulated in both Zheng Taizhi 3 and SP19 under HTS, and one gene encoding *AUX1* was specifically up-regulated in heat-sensitive SP19. In Zheng Taizhi 3, five genes encoding *SAUR* were up-regulated, and nine were down-regulated. One gene encoding *AUX1* was up-regulated and one was down-regulated. Three genes encoding *AUX/IAA* were up-regulated, and three were down-regulated. In SP19, six genes encoding *SAUR* were up-regulated, and five genes were down-regulated. Two genes encoding *AUX/IAA* were up-regulated, and two were down-regulated (Figure 7, Table S5). *SAUR*, as a rapid response gene induced by auxin, plays a crucial role in plant growth by regulating the expression of other related genes [39]. According to reports, the *SAUR* gene is induced in stress-tolerant varieties and inhibited under abiotic stress [40], and these changes may be closely associated with differences in thermotolerance.

Abscisic acid (ABA) is a stress hormone closely related to plant heat resistance, which is involved in plant physiological processes and protects plants from high-temperature damage [4]. The ABA signaling pathway is composed of many regulatory genes and turnover mechanisms related to adaptation to environmental changes. Many studies have shown that the three core parts of the ABA signal transduction pathway and the downstream central part of the ABA response element binding factor (ABF) signal transduction are regulated by high temperatures [41,42]. ABA signaling involves PYR/PYL/RCAR, Protein phosphatase 2C (PP2C), and Serine/threonine protein kinase (SnRK2) pathways. Eleven DEGs were found to be involved in the ABA signal pathway in the two sesame varieties. Among them, three *PYR/PYL* genes were up-regulated, two *PP2C* genes were up-regulated, and one *PP2C* gene was down-regulated in heat-sensitive SP19; additionally, one *ABF-encoding* gene was down-regulated. In contrast, in the Zheng Taizhi 3, one *PYR/PYL-encoding* gene was up-regulated, and two genes were down-regulated; two of the four genes encoding *PP2C* were up-regulated, and two genes were down-regulated; and one gene encoding *ABF* was down-regulated (Figure 7, Table S4). These findings showed that the ABA signal transduction pathway plays a vital, important role in the response of sesame to HTS and regulates the expression of downstream ABA-responsive genes. At the same time, most DEGs related to the ABA signal transduction pathway showed similar expression patterns in these two genotypes, indicating that the mechanisms through which ABA mediates the HTS response in sesame were conserved.

Most DEGs related to the BR and auxin signal transduction pathway showed similar expression patterns in these two genotypes, indicating that the mechanisms through which BR and auxin mediate the HTS response in sesame were conserved.

## 5. Conclusions

**Figure 8:**
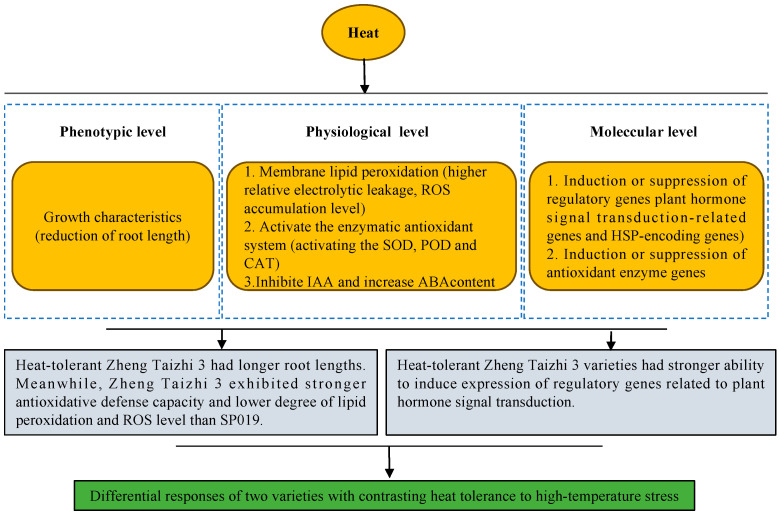
Model of the differential responses of two sesame cultivars with different heat tolerances under high-temperature stress found by comparative physiological and transcriptome analysis.

In this study, the thermotolerance in root growth during the germination of two sesame varieties was compared and analyzed based on physiological and transcriptomic analyses. As shown in Figure 8, the results showed that Zheng Taizhi 3 is more tolerant to HTS than SP19 and demonstrated the following aspects: (1) Physiologically, Zheng Taizhi 3 showed a stronger antioxidant defense ability, and the degree of membrane lipid peroxidation was lower. (2) Zhengtaizhi 3 exhibits more active gene expression regulation of the phenylpropanoid biosynthesis pathway, and the up-regulation of multiple key genes in Zhengtaizhi 3 is more pronounced, which may contribute to its stronger heat tolerance. (3) The HSP genes in both varieties are up-regulated, which is beneficial for combating a high-temperature stress environment. (4) In addition, Zhengtaizhi 3 had a stronger ability to induce the expression of regulatory genes related to plant hormone signal transduction, and exhibited a more refined regulation of auxin (IAA) and abscisic acid (ABA) signal transduction, which aids in maintaining normal growth and development under high-temperature stress. In the two sesame varieties, these genes have similar expression patterns and exhibit a certain degree of conservatism. Identifying these key candidate genes could contribute to the future application of sesame genetic improvement programs. However, the molecular regulation of heat-resistance-related genes in sesame remains unclear, and further investigation and verification are required.

## Data Availability

The data used to support the funding of this study are available from the corresponding author upon request.

## Supplementary Materials

The following supporting information can be downloaded at: https://www.mdpi.com/article/10.3390/cimb46120794/s1: Figure S1. Principal components analysis (A) and correlation heatmap (B) of the gene expression profiles in response to high-temperature stress. Figure S2. Validation of RNA sequencing data using qRT-PCR. Data are expressed as average ± SD, *n* = 3, and significances between treatments at *p* < 0.05 are indicated by different letters over columns (Duncan’s test). Figure S3. GO analysis of DEGs in different groups. Table S1. The expression pattern of DEGs involved in antioxidation. Table S2. The expression pattern of DEGs involved in phenylpropanoid biosynthesis. Table S3. The expression pattern of HSP-encoding genes. Table S4. The expression pattern of DEGs involved in plant hormone signal transduction. Table S5. List of primers used in this study for qRT-PCR.
