# Supplementary material for: Comprehensive Transcriptomic and Physiological Insights into the Response of Root Growth Dynamics During the Germination of Diverse Sesame Varieties to Heat Stress
Source: Curr Issues Mol Biol. 2024 Nov 22;46(12):13311–27. doi: 10.3390/cimb46120794 (PMC11727563; doi:10.3390/cimb46120794)
Supplement: Supplementary file 1 [file cimb-46-00794-s001.zip › Supplemental figure-new(1).pdf]

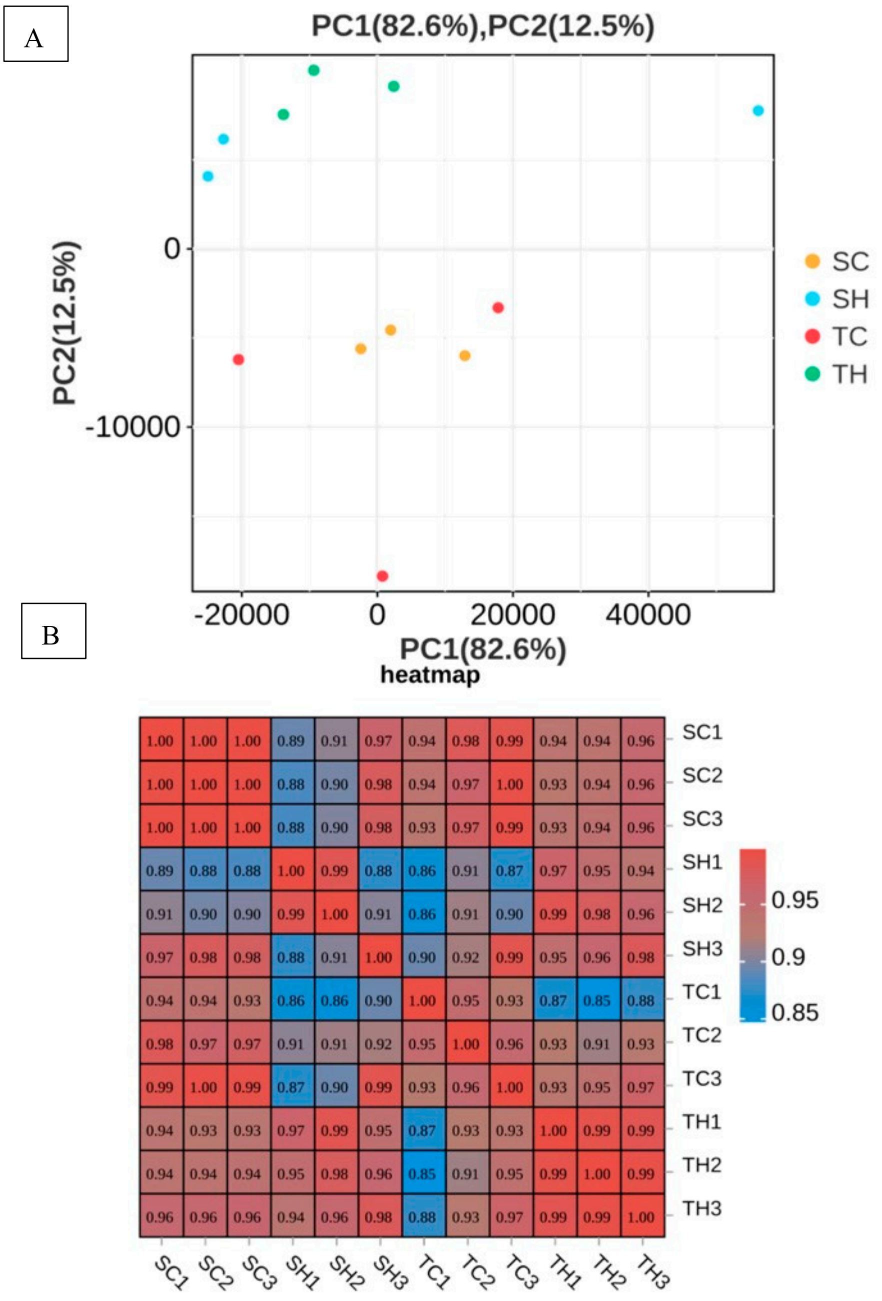

Figure S1. Principal components analysis (A) and correlation heatmap (B) of the gene expression profiles in response to high temperature stress.

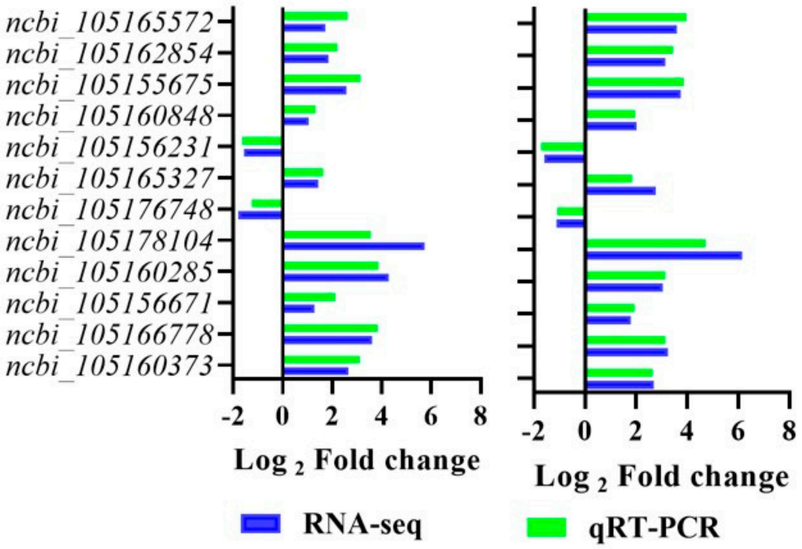

Figure S2. Validation of RNA sequencing data using qRT-PCR.

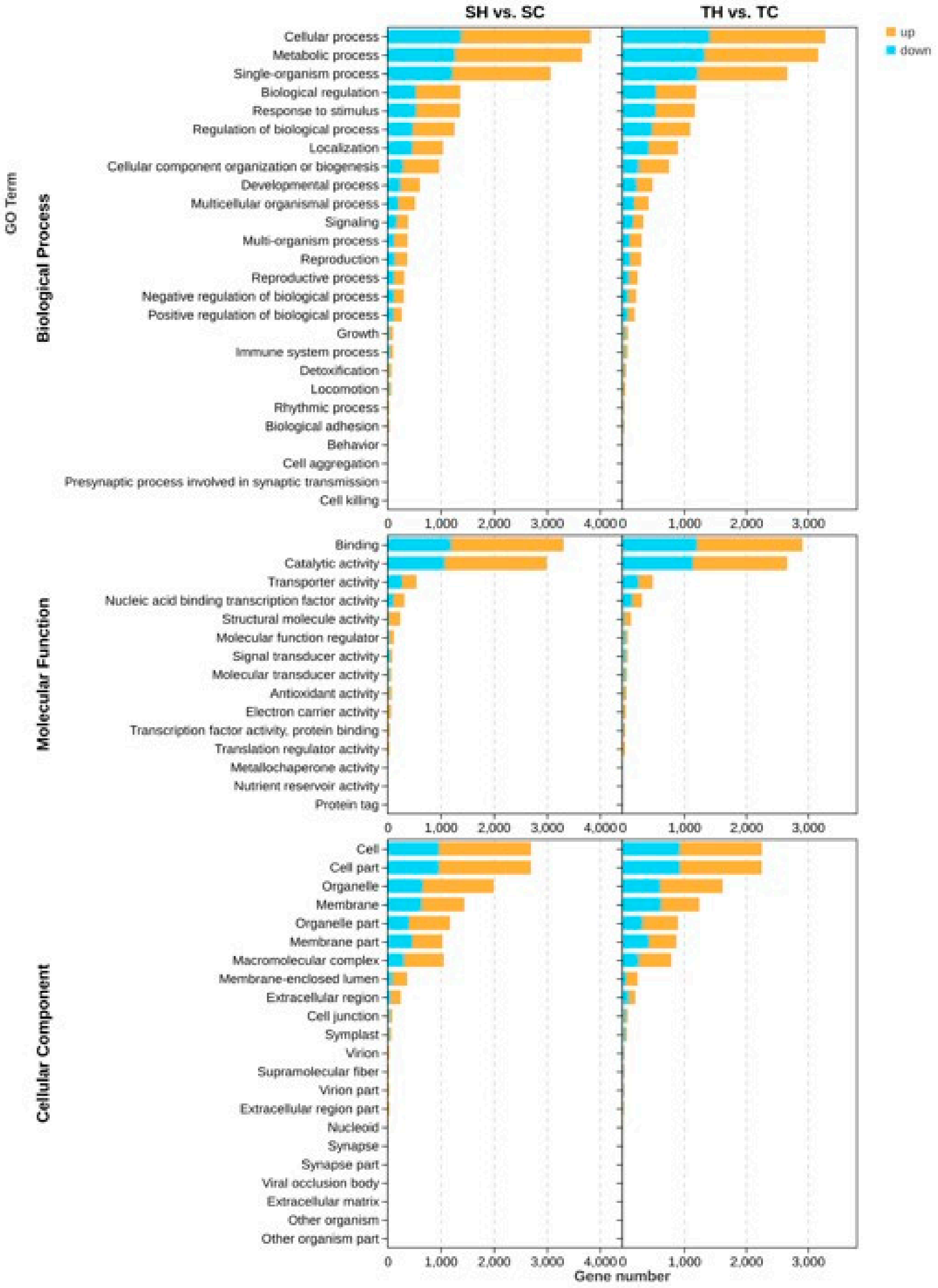

Figure S3. GO analysis of DEGs in different groups.
